# Assessment of functional performance in self-rectifying passive crossbar arrays utilizing sneak path current

**DOI:** 10.1038/s41598-024-74667-z

**Published:** 2024-10-21

**Authors:** Ziang Chen, Xianyue Zhao, Christopher Bengel, Feng Liu, Kefeng Li, Stephan Menzel, Nan Du

**Affiliations:** 1 Institute for Solid State Physics, Friedrich Schiller University Jena, Helmholtzweg 3, 07743 Jena, Germany; 2 Department of Quantum Detection, Leibniz Institute of Photonic Technology (IPHT), Albert-Einstein-Strasse 9, 07745 Jena, Germany; 3 Institute for Electronic Materials 2, RWTH Aachen University, Sommerfeldstrasse 18/24, 52074 Aachen, Germany; 4 Peter Grünberg Institut (PGI-7), Forschungszentrum Juelich GmbH, Wilhelm-Johnen-Strasse, 52428 Jülich, Germany

**Keywords:** Passive crossbar array, Self-rectifying memristive devices, Sneak path current, Read margin, Negative rectification factors, Structural properties, Electronic properties and materials

## Abstract

Self-rectifying memristive devices have emerged as promising contenders for low-power in-memory computing, presenting numerous advantages. However, characterizing the functional behavior of passive crossbar arrays incorporating these devices remains challenging due to sophisticated parasitic currents stemming from rich memristive dynamic behavior. Conventional methods using read margin assessments to evaluate functional behavior in passive crossbars are hindered by the voltage divider effect from the pull-up resistor. In this study, we propose a novel performance metric, $\Delta$SC, harnessing sneak path currents to assess functional behavior. Through the application of a pair of negative rectification factors, $\text {RF}_\text {n, L}$ and $\text {RF}_\text {n, H}$, we comprehensively delineate dynamic rectification behavior in both positive and negative bias regimes, as well as in low-resistance state and high-resistance state, deviating from conventional metrics such as on/off ratios, nonlinearity, and rectifying factors. Notably, $\Delta$SC provides a quantitative evaluation of the interaction between sneak path currents and read margin, demonstrating its efficacy and addressing a pivotal research gap in the field. For instance, employing self-rectifying BiFeO$_3$ memristive cells featuring $\text {RF}_\text {n, L}$ = 1.22E3 and $\text {RF}_\text {n, H}$ = 9.27, we showcase the successful functional performance of a passive crossbar array, achieving $\Delta$SC < 2.19E−2, while ensuring a read margin > 0.

## Introduction

**Fig. 1:**
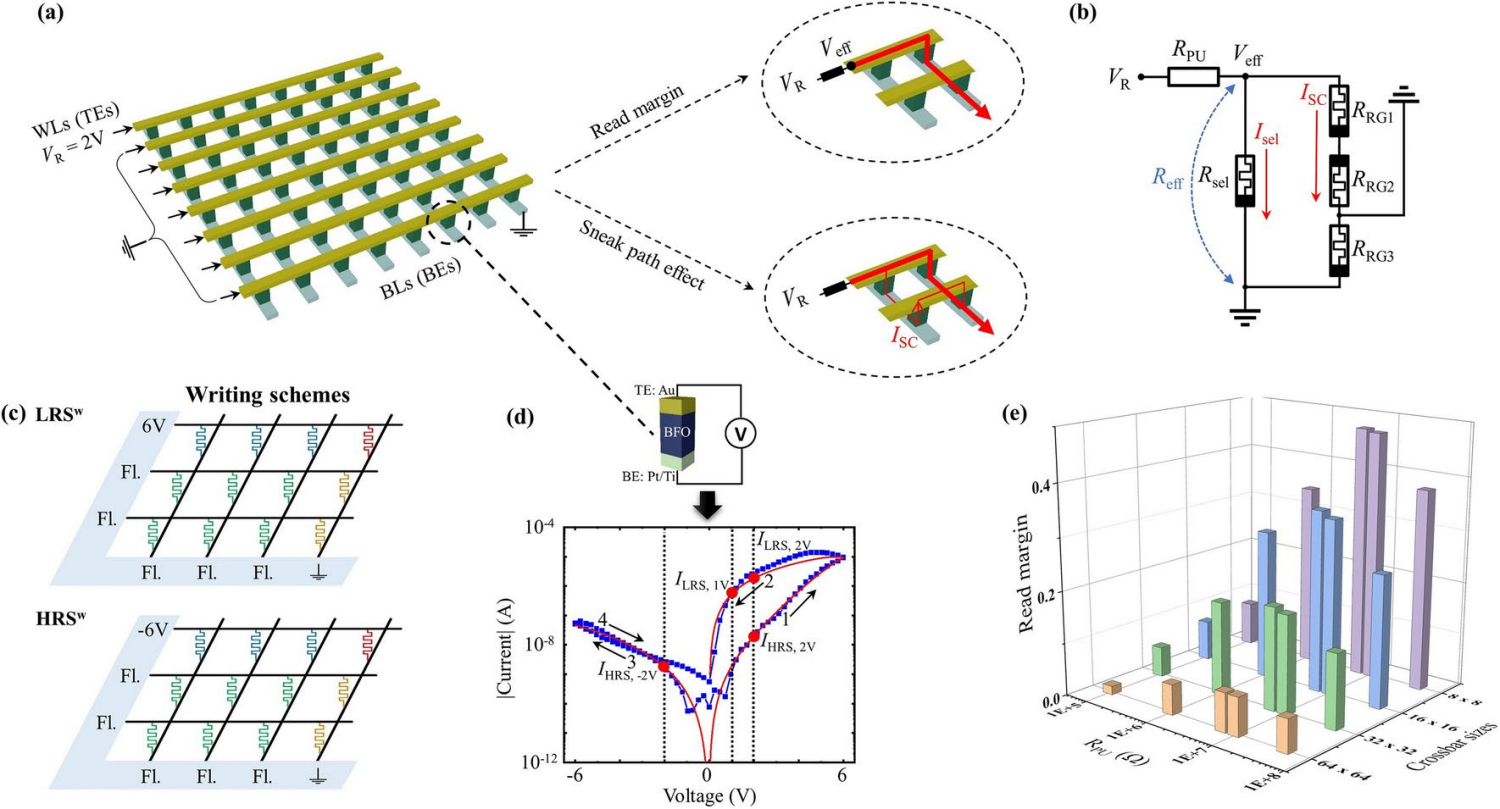
(**a**) Passive crossbar topology and voltage distribution in the reading scheme. Insets demonstrate the intrinsic relationship between RM and SC in passive crossbar: the RM can be applied to identify the valid functional behavior of a passive crossbar array, and the RM is affected by the SC. (**b**) Corresponding equivalent circuit of passive crossbar array in the reading scheme. (**c**) Demonstration of LRS-(LRS$^w$) and HRS-(HRS$^w$) writing schemes. (**d**) Simulated (in red) and experimental (in blue) $I-V$ characteristics of BiFeO$_3$ memristive cell. Inset shows the schematic sketch of the Au-BFO-Pt/Ti structure for the BFO memristor. (**e**) Evaluation of RM with respect to different values of pull-up resistance and crossbar sizes.

Emerging memristive crossbar technology^1,2^ has garnered significant attention due to its multifunctional advantages, including nonvolatility and reconfigurability, making it a compelling candidate for memory-centric computing hardware platforms^3,4^. However, this technology faces substantial challenges stemming from sneak path issues, where parasitic currents flow through unselected cells, resulting in a limited memory window for selected cells and operational failures^5–7^. While solutions like the one-selector-one-resistor (1S1R)^8–10^ or one-transistor-one-resistor (1T1R)^11–13^ configurations effectively mitigate parasitic currents by incorporating a selector or transistor beneath each memristive cell, they come at the cost of increased area and power consumption. To address this trade-off between sneak path current (SC) and memristive system efficiency, passive crossbar configurations based on self-rectifying memristive devices^4,14,15^ have emerged as a promising alternative, offering substantial suppression of parasitic current, ultra-high integration density, and low power consumption.

In the realm of current state-of-the-art research, the read margin (RM) has firmly established itself as a widely accepted metric for appraising the functional behavior of passive crossbar arrays. However, previous studies have predominantly centered on exploring the relationship between RM and the dynamic switching characteristics of self-rectifying memristive cells, often focusing on only one or two parameters such as the on/off ratio, nonlinearity, or rectifying factor. This limited scope has failed to offer a comprehensive characterization of self-rectifying behavior across both the high-resistance state (HRS) and low-resistance state (LRS). Furthermore, when RM evaluations have been approached analytically, they have frequently considered only the on/off ratio, inadvertently disregarding the nuanced dynamical switching behaviors, especially in the context of reversed bias. Additionally, the precise determination of RM heavily hinges on the inclusion of a precisely defined pull-up resistor interconnected in series with the crossbar, a factor that renders RM results acutely susceptible to variations in pull-up resistance values. Moreover, the sophisticated sneak path effect, stemming from the richly dynamic behavior exhibited by self-rectifying memristive cells, poses formidable challenges in the quantitative exploration of the interplay between sneak path current and RM within passive crossbar arrays, giving rise to a significant research gap. Understanding this interrelationship is pivotal for effectively achieving the desired functional behavior in passive crossbars.

In this study, we present an in-depth analysis of the sneak path effect within passive crossbars, contrasting it with corresponding assessments of RM. Building upon this in-depth investigation, we introduce a novel performance metric, $\Delta$SC, which leverages sneak path currents to gauge the functional behavior of passive crossbar arrays. The unique aspect of $\Delta$SC lies in its incorporation of newly proposed negative rectification factors, $\text {RF}_\text {n, L}$ and $\text {RF}_\text {n, H}$, applicable to both the LRS and HRS. These factors provide a comprehensive characterization of the self-rectifying behavior in memristive cells, offering sufficient information for accurately characterizing the sneak path features in passive crossbars that utilize these self-rectifying cells. Using self-rectifying BiFeO$_3$  (BFO) memristive cells as an example, we modulate the dynamic switching behaviors of these devices to showcase the interplay between $\Delta$SC and RM. This demonstration effectively validates the utility of $\Delta$SC and establishes a direct quantitative relationship between sneak path current and RM, thereby closing a critical research gap in the field.

## Results and discussion

### Evaluation of functional performance in passive crossbar arrays

Passive crossbar arrays by using self-rectifying memristive cells, in contrast to the 1T1R topology, eliminating the need for active electronic components at each junction. These arrays offer numerous advantages, including high integration density, low power consumption, scalability, and simplified peripheral design. The utilization of self-rectifying memristive cells in passive crossbar arrays has been the subject of extensive research over the past decade, as evidenced by studies such as^14–16^. Figure 1a provides an illustration of a passive crossbar array based on self-rectifying memristive cells, featuring horizontal wordlines (WLs) as shared top electrodes (TEs) and vertical bitlines (BLs) as shared bottom electrodes (BEs).

To evaluate the functional performance of a passive crossbar array, the RM, as depicted in Fig. 1a, serves as a common metric. The upper inset to the right of the passive crossbar elucidates that the RM quantifies the variation in effective voltage drops ($V_\text {eff}$) across the crossbar array for a selected memristive cell, operating in either the LRS or HRS, while a reading voltage $V_\text {R}$ is applied. Conversely, the sneak path current ($I_\text {SC}$), a primary factor diminishing RM performance in passive crossbars, is visually explicated in the lower inset, demonstrating the flow of current through unintended inactive pathways running parallel to the desired path when the memristive crossbar array is active. The corresponding equivalent circuit is illustrated in Fig. 1b.

For assessing RM, a pull-up resistor $R_\text {PU}$ is introduced in series with the passive crossbar, as illustrated in Fig. 1b. It is important to note that, for an accurate characterization of RM, the passive crossbar arrays were subjected to worst-case scenarios^4,17^. In this context, a specific selected cell, denoted in red in Fig. 1c, is systematically toggled between the LRS and HRS, while the remaining unselected inactive cells are maintained in the opposite states.

RM assessment entails two distinct writing schemes under worst-case conditions: LRS-writing (LRS$^w$) and HRS-writing (HRS$^w$). In each writing scheme, a sequence of two steps is executed for the passive crossbar array: initialization and writing steps. In the LRS$^w$ (HRS$^w$) scheme, all memristive cells are initially set to HRS (LRS) in the initialization step. Subsequently, in the writing phase, the selected cell exclusively transitions to LRS in the LRS$^w$ scheme (or HRS in the HRS$^w$ scheme), while the unselected cells remain in HRS (LRS). Figure 1c illustrates four distinct regions established during the LRS$^w$ and HRS$^w$ writing schemes: the selected cell, the semi-selected Region 1 (RG1, marked in blue), the inactive Region 2 (RG2, marked in green), and the semi-selected Region 3 (RG3, marked in yellow). Followed by the writing scheme, the reading step is applied for reading out the actual resistance state of the selected cells. In the reading phase, the effective voltage values across the selected cell in LRS and HRS are recorded, and RM is computed as the difference between these effective voltages, defined as follows:

1 $$\begin{aligned} \text {RM} = \frac{V_\text {eff,HRS}-V_\text {eff,LRS}}{V_\text {R}}, \end{aligned}$$

where $V_\text {eff,HRS}$ and $V_\text {eff,LRS}$ represent the effective voltages of the selected cell in the HRS or LRS, corresponding to the application of HRS$^w$ and LRS$^w$ writing schemes, respectively, while $V_\text {R}$ denotes the reading bias. Under worst-case scenario, If RM > 0, it signifies that the selected cell within the passive crossbar array can effectively distinguish between the LRS and HRS states. This indicates the presence of a distinct reading window for the selected cell, independent of the initial resistance states of all the cells within the passive crossbar, ultimately indicating the valid functional behavior of the passive crossbar.

Table 1 provides a systematic summary of previous studies that have individually investigated the influence of various relevant parameters of self-rectifying memristive cells on RM of passive crossbar arrays. Based on the existing studies listed in Table 1, we have categorized prior research on RM evaluation and the sneak path effect in 1R passive crossbar arrays into two main categories: simulation-based studies utilizing analytical solutions and memristor models. Note that, if the parameter values are denoted within a range in Table 1, it signifies that the parameter was subject to varying numerical adjustments to examine its impact on the RM. The experimental studies involving RM evaluation, conducted on fabricated 1R passive crossbars^4^, primarily center on the discussion of RM while progressively expanding the crossbar size, with less comprehensive consideration of the broader impact on other relevant parameters. Consequently, these studies have not been incorporated into Table 1.

**Table 1:** A summary of prior investigations pertaining to the impact of various parameters on the RM within 1R passive crossbar arrays. The considered parameters encompass on/off ratio, nonlinearity (NL), rectification factor (RF), crossbar size, line resistance ($R_\text {Line}$), and pull-up resistance ($R_\text {PU}$). Note that, RF is corresponding to rectification factor in LRS, $\text {RF}_\text {n, L}$, in this work. In our work, in additional to the listed parameters, we have further studied $\text {RF}_\text {n, H}$ = 9.27E−1–9.27E1, as one of the relevant parameters for RM evaluation.

Works	Methods (software)	M classification (M stack)	Relevant parameters for RM evaluation			$R_\text {Line}$ ($\Omega$)	$R_\text {PU}$ ($\Omega$)	Crossbar sizes
			On/off	NL	RF			
A. Flocke, 2007^18^	Analyticalsolution (–)	– (–)	10$^1$–10$^6$	–	–	20	–	$1 \times 1$–$100 \times 100$
A. Flocke, 2008^19^	Analytical solution (–)	Bipolar (Pt/TiO$_2$/Ti/Pt)	10$^1$	1–10$^2$	–	15	–	10 $\times$ 10–120 $\times$ 120
E. Linn, 2010^20^	Analytical solution (–)	CRS (Pt/SiO$_2$/GeSe/Cu)	10$^1$–10$^5$	–	–	–	10$^3$–$2 \times 10^3$	$2 \times 2$–1E5 $\times$ 1E5
A. Ciprut, 2016^21^	Analytical solution (SPICE)	– (–)	10$^1$–10$^4$	10$^1$–10$^5$	–	–	–	$200 \times 200$
A. Chen, 2017^22^	Analytical solution (HSPICE)	– (–)	10$^1$	0–10$^2$	–	–	0-$R_\text {LRS}$	$80 \times 80$–$320 \times 320$
R. Ni, 2021^23^	Analytical solution (–)	Bipolar (Pt/TaO$_x$/Ta)	10$^4$	0, 10$^5$	$9 \times 10^4$	–	$4.7 \times 10^6$	$1 \times 1$–2E4 $\times$ 2E4
K. Zhang, 2022^24^	Analytical solution (–)	Bipolar (Al/AlN/W)	6.1 $\times$ 10$^3$	–	0, $2.6 \times 10^3$	–	$R_\text {LRS}$	$1 \times 1$–3E4 $\times$ 3E4
J. Zhou, 2014^25^	Memristor model (HSPICE)	Bipolar (-)	10$^2$–10$^5$	–	–	5	10$^1$–10$^3$	$8 \times 8$–$512 \times 512$
Y. Gao, 2016^26^	Memristor model (Cadence)	Bipolar (–)	10$^3$	0.5–8	10$^3$–10$^6$	5–320	$1.6 \times 10^7$	$4 \times 4$–$128 \times 128$
C. Li, 2019^27^	Memristor model (SPICE)	Bipolar (p-Si/SiO$_2$/n-Si)	10$^4$	–	10$^5$	0–10$^3$	–	$3 \times 3$–1E3 $\times$ 1E3
T. Kim, 2021^28^	Memristor model (SPICE)	Bipolar (Cu/TiO$_x$/Al)	–	–	3.8 $\times$ 10$^2$	–	10$^3$–10$^7$	16 $\times$ 16–256 $\times$ 256
T. Kim, 2021^28^	Memristor model (SPICE)	Bipolar (Al/TiO$_x$/Al)	–	–	1.5	–	1–10$^4$	16 $\times$ 16–256 $\times$ 256
Z. Chen, 2022^29^	Memristor model (Cadence)	Bipolar (Au/BiFeO$_3$/Pt/Ti)	131.5	3.5	1.22 $\times$ 10$^2$–1.22 $\times$ 10$^4$	10$^1$	6.5 $\times$ 10$^6$	4 $\times$ 4
Our work	Memristor model (Cadence)	Bipolar (Au/BiFeO$_3$/Pt/Ti)	24.2–243.7	2.0–8.0	1.22 $\times$ 10$^2$–1.22 $\times$ 10$^4$	10$^1$	10$^5$–10$^8$	8 $\times$ 8–128 $\times$ 128

Simulation-based studies employing analytical solutions for evaluating are presented in the upper section of Table 1, encompassing the first seven rows, which leverages Kirchhoff’s law to simplify the comprehensive crossbar array. In contrast, simulation-based studies utilizing memristor models for RM evaluation (in the lower section of Table 1, covering the last seven rows) typically involve the creation of simulation models tailored to match the device-specific $I-V$ characteristics of memristive devices. These memristive device simulation models are then interconnected electrically to form a crossbar array, and simulation is performed using tools such as SPICE or Cadence. It’s worth noting that, in this context, only memristor mathematical models have been considered for RM evaluation. Physical compact models are not required, as the memristor mathematical models accurately capture the switching behavior of memristive passive crossbar arrays without the need for a physical background.

As aforementioned, the simulation-based studies employing analytical solutions rely on Kirchhoff’s law to amalgamate regions within the passive crossbar array that experience identical voltage differentials. This process effectively simplifies a full-scale crossbar array into a 2 $\times$ 2 equivalent circuit for rough simulation purposes. While this approach permits the simulation of large-scale crossbar arrays, with dimensions reaching as expansive as 1E5 $\times$ 1E5^20^, it typically sacrifices simulation precision compared to the memristor model methodology. In our work, summarized in the last row of Table 1, we employ simulation-based studies utilizing memristor models, which can more accurately capture the dynamical behavior of memristive devices electrically. Compared to other simulation-based studies utilizing memristor models, which often focus on only one or two relevant parameters^25,27–29^, our study provides a more comprehensive examination. In Reference^26^, the independence of RM on various parameters, such as nonlinearity, rectification factors, and crossbar sizes, was investigated. In contrast, our study goes beyond these parameters, encompassing the influence of on/off ratio and $R_\text {PU}$, which were not considered in^26^. Building upon these findings, in our work, we explore the interactions between RM and sneak path current, subsequently developing a novel evaluation methodology for assessing RM through the analysis of sneak path current.

#### Passive crossbar array based on BFO memristive devices

In this study, we employed self-rectifying BFO memristive cells to construct a passive crossbar array, driven by several critical factors. Our ability to fabricate physical BFO memristors enables direct experimental validation and meaningful comparison between simulations and real devices, ensuring the reliability and relevance of our findings. BFO memristors exhibit superior performance metrics, such as outstanding retention, high uniformity, and endurance up to 10^6^ cycles, making them ideal for comparative studies^30–32^. Our decade-long expertise in electric field-controlled ion-induced switching in BFO-based memristors, including BFO, $\text {BiFeTiO}_3$ (BFTO), and $\text {BiFeTiO}_3/\text {BiFeO}_3$ (BiBFO) variants, allows us to achieve diverse hysteresis characteristics, which are crucial for exploring novel applications^30,33–41^. For instance, the BFO-based memristor, owing to its remarkable features, exhibits the potential to function as an artificial synapse in neuromorphic computing^35,37^, as a fundamental element for reconfigurable logic gate in in-memory computing^41,42^, and for implementing cryptofunctions for studying security vulnerability of memristive devices^38–40^.

As shown in inset in Fig. 1d, the BFO memristor consists of polycrystalline BFO thin film, which is sandwiched between an Au TE and a Pt/Ti BE adhered onto a SiO$_2$/Si substrate. Under the triangle shaped ramping bias with an amplitude of $|V_\text {W}|$ = 6 V, the experimental $I-V$ characteristics of BFO memristor are recorded and demonstrated in Fig. 1c (marked in blue), which exhibit the bipolar rectifying switching dynamics in BFO cell. The BFO memristive device can be switched between the LRS and the HRS by applying writing pulses with opposite polarity. During a SET pulse, a positive voltage (+$V_\text {W}$ = 6 V) is applied to the TE while the BE is grounded, leading to the BFO memristor being in the LRS. Similarly, a RESET pulse involves applying a negative voltage ($-V_\text {W}$ = -6 V) to the TE with the BE grounded, resulting in HRS. The resistance states, LRS and HRS, can be determined at a small reading bias $V_\text {R}$ of 2 V. The mechanism of the bipolar resistive switching observed in BFO memristor can be explained by the modification of the Schottky barrier at the BFO-Pt bottom interface by the drift of charged oxygen vacancies under applied large electric fields during the writing step^36^. In this work, in order to study the switching dynamic dependence, we utilized BFO Verilog-A model for constructing passive crossbar array in Cadence Virtuoso. The mathematical equations for the BFO Verilog-A model, along with the corresponding parameter values, can be found in Supplementary Fig. S1. The $I-V$ characteristics of the simulated BFO memristive device, shown in Fig. 1c (red line), exhibit consistency with experimental results.

#### Evaluation of functional performance by varying $\text {R}_\text {PU}$

Evaluation of the functional performance of a passive crossbar through RM necessitates the connection of $R_\text {PU}$ in series with the passive crossbar. RM assessment relies on the voltage divider effect occurring between the passive crossbar and $R_\text {PU}$. Here we firstly examine the influence of RM while systematically varying the value of $R_\text {PU}$. In this study, during the assessment of functional performance in passive crossbars utilizing self-rectifying BFO memristors by varying different parameters, we employed floating writing scheme and One Wordline Pull-Up (OneWLPU) reading scheme. In floating writing scheme, the SET (RESET) bias was applied to the selected WL while grounding the selected BL (Fig. 1c) during the LRS$^w$ (HRS$^w$) writing phases, leaving the unselected WLs/BLs floating. Furthermore, OneWLPU reading scheme has been selected in reading step because it is widely used in practical testing of passive crossbars in numerous studies, due to its ability to effectively suppress sneak currents^26,43^. As illustrated in Fig. 1a, to implement the OneWLPU reading scheme, a reading bias of $V_\text {R}$ = 2 V was applied simultaneously to the selected WL in the LRS (HRS), while the unselected WLs were grounded. The selected BL was grounded, while the unselected BLs were left floating. During this phase, parasitic currents flowed through the memristive cells from the TE to the BE in RG1 and from BE to TE in RG2 due to the grounded WLs in RG2. In RG3, where both TE and BE were grounded, no sneak current was observed. The illustrative and corresponding equivalent circuit for the OneWLPU reading scheme are depicted in Fig. 1a and b, respectively.

Figure 1e demonstrate the evaluation of RM dependence on $R_\text {PU}$ within the range of 1.0E5 $\Omega$ to 5.0E7 $\Omega$, considering various crossbar sizes ranging from 8 $\times$ 8 up to 64 $\times$ 64. The results reveal that $R_\text {PU}$ plays a crucial role and strongly influences the RM performance. Notably, across all crossbar sizes, an optimal value of $R_\text {PU}$ at 6.5E6 $\Omega$ exhibits the best RM performance, indicating the ideal reading performance for the crossbar topology across different sizes. Therefore, the $R_\text {PU}$ value of 6.5E6 $\Omega$ is selected for further simulation implementation in the subsequent sections of this study. It is important to highlight that in the state-of-the-art work^18,26^, the computation of $R_\text {PU}$ is done using the equation $R_\text {PU}$ = $\sqrt{R_\text {HRS}\cdot R_\text {LRS}}$, where $R_\text {HRS}$ and $R_\text {LRS}$ represent the resistance values of the HRS and LRS in the corresponding memristive cells. By taking the BFO $I-V$ characteristics shown in Fig. 1c and considering the values of $R_\text {HRS}$ = 4.5E7 $\Omega$ and $R_\text {LRS}$ = 0.9E6 $\Omega$ at a reading bias of 2 V, we calculated the value of $R_\text {PU}$ to be 6.5E6 $\Omega$. This result is consistent with the simulation observations presented in Fig. 1e and agrees with the results reported in the earlier state-of-the-art work^18,26^. These consistent results validate the accuracy of RM evaluation on passive crossbar in this work.

**Fig. 2:**
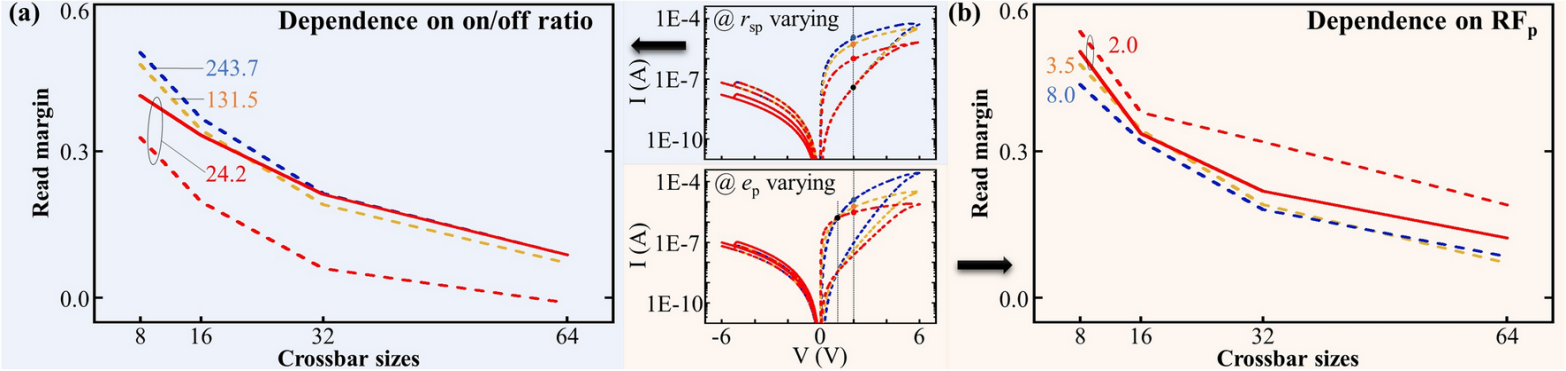
Evaluation of RM with respect to crossbar sizes up to 64 $\times$ 64 in dependence on the modification of (**a**) the on/off ratios (243.7 (blue dash line)/131.5 (orange dash line)/24.2 (red dash line)) by varying $r_\text {sp}$ (5.0E−3 $\Omega$/5.0E−2 $\Omega$/5.0E−1 $\Omega$) and (**b**) the nonlinearity $\text {RF}_\text {p}$ (8.0 (blue dash line)/ 3.5 (orange dash line)/ 2.0 (red dash line)) by varying $e_\text {p}$ (3.0 / 1.8 / 1.0). $k_\text {n}$ is kept as 1.0E−7 V. Insets demonstrate corresponding $I-V$ characteristics (**a**) by varying $r_\text {sp}$ and (**b**) by varying $e_\text {p}$. The solid red lines illustrate (a) a slight decrease in leakage current achieved by reducing $k_\text {n}$ from 1.0E−7 V to 2.5E−8 V, and (**b**) a slight increase in leakage current achieved by increasing $k_\text {n}$ from 1.0E−7 V to 1.5E−7 V, while maintaining (**a**) a constant on/off ratio of 24.2 ($r_\text {sp}$ = 5.0E−1 $\Omega$) and (**b**) $\text {RF}_\text {p}$ = 2.0 ($e_\text {p}$ = 1.0). This slight change of $k_\text {n}$ has a significant effect on the evaluation of RM, indicating that the on/off ratio and $\text {RF}_\text {p}$ cannot be used as reliable parameters for assessing the functional performance of passive crossbars.

In this study, we utilize Cadence Virtuoso for mixed-signal circuit simulation, which offers higher fidelity and the capability to conduct large-scale simulations, surpassing the limitations of existing analytical solutions using Matlab and other methods^44–46^. However, it is important to note that the inherent non-idealities, such as cycle-to-cycle (C2C) and device-to-device (D2D) variations, as well as endurance issues, which are critical in the practical implementation of memristive crossbar arrays, are not specifically addressed within the scope of this work. During simulation, the line resistance has been configured to a reasonable value of 10 $\Omega$ in this work. Given the high resistance values characteristic of BFO memristors, typically surpassing the M$\Omega$ range, line resistance has a negligible impact on passive crossbar arrays constructed with BFO memristors. According to our simulations, line resistance would only affect the RM when it exceeds 10 K$\Omega$ in a 64 $\times$ 64 passive crossbar array. Therefore, within the scope of this work, line resistance has not been a subject of investigation.

#### Evaluation of functional performance by varying on/off ratio and nonlinearity

Two key metrics are commonly used to characterize the self-rectifying behavior of memristive devices: the on/off ratio and the nonlinearity (indicated as positive rectification factor $\text {RF}_\text {p}$ in this work). The on/off ratio is defined as the ratio between the current values through the memristive device in the LRS and HRS at the reading bias voltage $V_\text {R}$. The nonlinearity, on the other hand, is defined as the ratio between the current values through the memristive device in the LRS at the reading bias voltage $V_\text {R}$ and half of the reading bias voltage (1/2 $V_\text {R}$). To illustrate this, consider the self-rectifying BFO memristor shown in Fig. 1d. The on/off ratio and the $\text {RF}_\text {p}$ can be calculated as follows:

2 $$\begin{aligned} \text {On}/\text {off} = \frac{I_\text {LRS,2V}}{I_\text {HRS,2V}},\qquad \text {RF}_\text {p} = \frac{I_\text {LRS,2V}}{I_\text {LRS,1V}},\qquad \end{aligned}$$

where $I_\text {LRS,2V}$ and $I_\text {HRS,2V}$ denotes the current values at the reading bias of $V_\text {R}$ = 2 V through the BFO memristive device in the LRS and HRS, respectively. Additionally, $I_\text {LRS,1V}$ represents the current values at 1 V through the BFO memristive device in the LRS. It is important to note that in the BFO memristive cell, a lower $\text {RF}_\text {p}$ corresponds to a higher level of nonlinearity.

The self-rectifying switching dynamics can be modulated by adjusting the parameter values of the BFO model: decreasing $r_\text {sp}$ can effectively increase the current value of LRS, thus the on/off ratios can be increased as shown in the inset of Fig. 2a. On the other hand, by decreasing $e_\text {p}$, the current value under maximum positive writing bias is decreased, thus $\text {RF}_\text {p}$ can be reduced as demonstrated in the inset of Fig. 2b.

The corresponding evaluated RM values in dependence of on/off ratio and $\text {RF}_\text {p}$ are illustrated in dash lines in Fig. 2a, b, respectively, with an increased crossbar size from 8 $\times$ 8 to 64 $\times$ 64. The decrease in the on/off ratio is accompanied by a gradual reduction in the RM of the 8 $\times$ 8 crossbar array, providing evidence for a decline in its performance (Fig. 2a). Furthermore, as the size of the crossbar array expands from 8 $\times$ 8 to 64 $\times$ 64, a consistent degradation in RM is observed (Fig. 2a), indicating that a decrease in the on/off ratio consistently leads to a reduction in RM. On the other hand, the gradual reduction in RM is observed in the 8 $\times$ 8 crossbar array with the enhancement of $\text {RF}_\text {p}$ (Fig. 2b). However, as the crossbar array size increases from 8 $\times$ 8 to 64 $\times$ 64, the impact of $\text {RF}_\text {p}$ on RM becomes erratic and unpredictable (Fig. 2b). This is evident from the intersecting orange ($\text {RF}_\text {p}$ = 3.5) and blue curves ($\text {RF}_\text {p}$ = 8), suggesting that $\text {RF}_\text {p}$ is not a conclusive parameter that affects RM.

Furthermore, it is important to note that a significant modification in the RM can be achieved by incorporating a minor adjustment to the leakage current in the reversed bias region while keeping the on/off ratio and $\text {RF}_\text {p}$ unchanged. This adjustment in the leakage current of the memristive cell is accomplished by slightly varying the value of $k_\text {n}$ in the BFO memristor model. In Fig. 2a, a decreasing adjustment of the leakage current, achieved by modifying the $k_\text {n}$ values from 1.0E−7 V to 2.5E−8 V, leads to a significant enhancement of the RM. This is evident from the deviation of the solid red curve from the baseline (dashed red curve). The intersection of the solid red curve with the dashed orange and blue curves further confirms the impact of this manipulation of the leakage current on the overall behavior of the crossbar array. Similarly, in Fig. 2b, an increasing adjustment of the leakage current by varying the $k_\text {n}$ value from 1.0E−7 V to 1.5E−7 V results in a substantial reduction in the RM (solid red curve), intersecting with the orange curve. These results suggest that the influence of leakage current on the RM can outweigh that of the on/off ratio and $\text {RF}_\text {p}$. It is evident that leakage current in the reversed bias region plays a more pivotal role in determining the self-rectifying behavior and affecting RM, which cannot be adequately captured by the on/off ratio and $\text {RF}_\text {p}$. This challenges the use of on/off ratio^27,47–49^ and nonlinearity^4,14,50,51^ as the sole parameters to characterize the self-rectifying behavior and evaluate the functional performance of a crossbar array, as proposed in most of the state-of-the-art works as cited here. For precise analysis of the interaction between RM and the sneak path effect, and for an accurate characterization of the functional performance of passive crossbars using sneak path current, it is imperative to introduce new parameters that specifically incorporate the characteristics of leakage current under reversed bias conditions.

#### Definition and performance evaluation using negative rectification factors

The limitation of using on/off ratio and $\text {RF}_\text {p}$ as sole parameters for evaluating functional behaviors in passive crossbar is due to their inability to capture the leakage current, which is a crucial feature of self-rectifying cells. Hence in this work, we propose the negative rectification factors in LRS and HRS, i.e. $\text {RF}_\text {n, L}$ and $\text {RF}_\text {n, H}$, which comprehensively captures the rectifying behaviors in both positive and negative bias regions of the memristive cell. For the BFO memristive cell, the negative rectification factors are determined as follows:

3 $$\begin{aligned} \text {RF}_\text {n, L} = \left| \frac{I_\text {LRS,2V}}{I_\text {HRS,-2V}} \right| , \qquad \text {RF}_\text {n, H} = \left| \frac{I_\text {HRS,2V}}{I_\text {HRS,-2V}} \right| ,\qquad \end{aligned}$$

where $I_\text {HRS,-2V}$ represents reading current at $V_\text {R}$ = -2V. As shown in Eq. (3), the parameter $\text {RF}_\text {n, L}$ represents the ratio of the LRS current in the positive voltage range to the leakage current in the negative voltage range. Similarly, $\text {RF}_\text {n, H}$ represents the ratio of the HRS current in the positive voltage range to the leakage current. The ratio between $\text {RF}_\text {n, L}$ and $\text {RF}_\text {n, H}$ corresponds to the on/off ratio. However, relying solely on the on/off ratio, as is the case in some previous studies in Table 1, proves inadequate for predicting the functional behavior of passive crossbars. This limitation arises from the omission of critical features within the reversed bias range as proven in the previous section. In this section, we advocate the use of negative rectification factors, incorporating both $\text {RF}_\text {n, L}$ and $\text {RF}_\text {n, H}$ into our performance analysis. This approach not only encompasses the on/off ratio and leakage current in reversed bias, but also provides a comprehensive characterization of the rectifying behaviors in both the LRS and HRS of the memristive cell. As a result, $\text {RF}_\text {n, L}$ and $\text {RF}_\text {n, H}$ provide accurate self-rectifying features in the cell, enabling RM evaluation along side sneak path current under both LRS$^w$ and HRS$^w$ writing schemes.

**Fig. 3:**
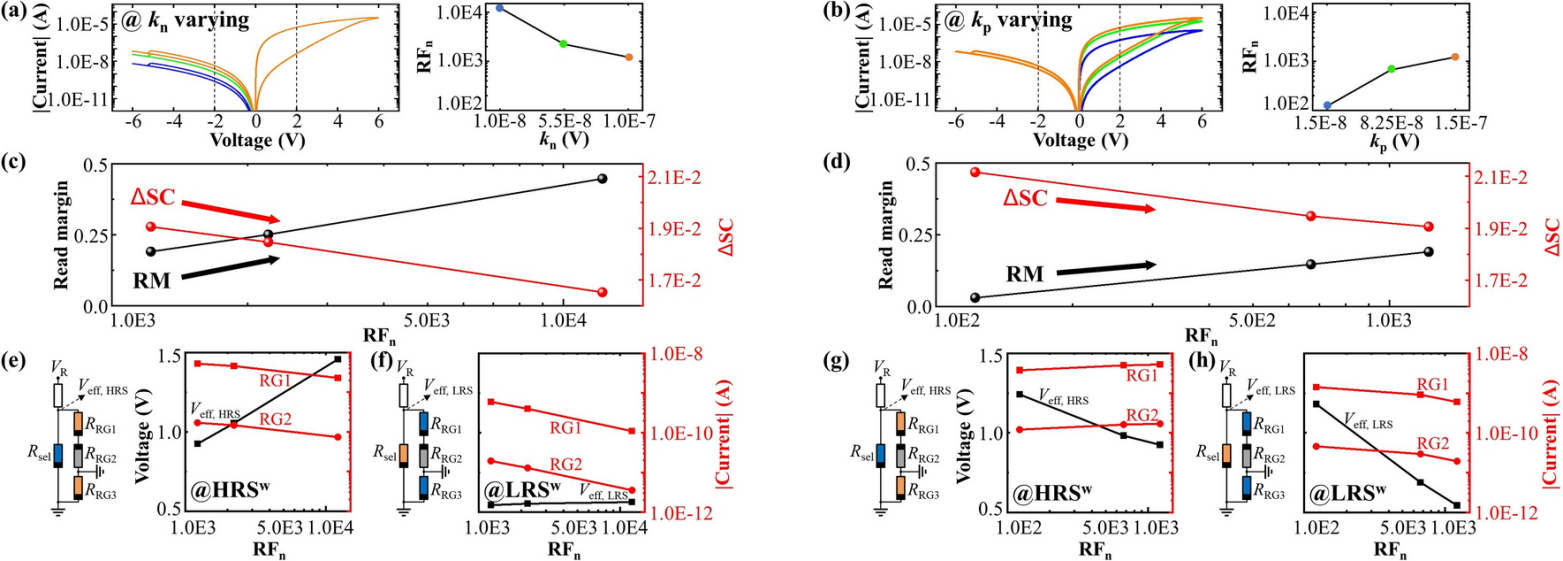
Demonstration of quantitative relationships between RM and SC with respect to $\text {RF}_\text {n}$. Illustration of $\text {RF}_\text {n}$ dependent $I-V$ characteristics (**a**) by varying $k_\text {n}$ and (**b**) by varying $k_\text {p}$ in the BFO model. The insets demonstrate recorded $\text {RF}_\text {n}$ in dependence of (**a**) $k_\text {n}$ and (**b**) $k_\text {p}$. Comparative evaluation of RM and $\Delta$SC with respect to $\text {RF}_\text {n}$ (**c**) by varying $k_\text {n}$ and (**d**) by varying $k_\text {p}$ in passive crossbar with size of 32 $\times$ 32. Demonstration of recorded effective voltages in dependence of $\text {RF}_\text {n}$ by varying $k_\text {n}$ in both (**e**) HRS$^w$ and (**f**) LRS$^w$, and by varying $k_\text {p}$ in (**g**) HRS$^w$ and (h) LRS$^w$, in comparison to recorded SC through individual memristive cell in RG1 and RG2. The insets demonstrate the corresponding equivalent circuits with marked initial resistive states in region cells during reading scheme (orange: LRS, blue: HRS, gray: reverse biased HRS). The line resistors in WLs and BLs are all determined as 10 $\Omega$ in all crossbar simulations.

It is important to note that $$\text {RF}_\text {n, L}$$ must be $$\gg$$ 1, indicating valid rectifying behavior in memristive devices for constructing a passive crossbar. On the other hand, $$\text {RF}_\text {n, H}$$ can be either larger or smaller than 1, which has a distinct impact on the sneak path effect and subsequently influences the RM. In general, a value of $$\text {RF}_\text {n, H}>$$ 1 is preferred as it signifies superior rectifying behavior in the memristive cell, leading to a larger RM compared to the case when $$\text {RF}_\text {n, H}<$$ 1. To evaluate the functional behavior of the passive crossbar array based on negative rectification factors, the influence of $$\text {RF}_\text {n, L}$$ and $$\text {RF}_\text {n, H}$$ needs to be considered separately. In this study, we specifically chose to vary $$k_\text {n}$$ / $$k_\text {p}$$ for modulating both negative rectification factors while keeping the on/off ratio at a constant value of 131.5 and the nonlinearity $$\text {RF}_\text {p}$$ = 3.5 unchanged. This approach allowed us to focus exclusively on examining the impact of negative rectification factors on the RM and the sneak path effect. Consequently, the ratio between $$\text {RF}_\text {n, L}$$ and $$\text {RF}_\text {n, H}$$, i.e. the on/off ratio of memristive cell, remains unchanged. Hence, in this work, to simplify the redundant expressions on $$\text {RF}_\text {n, L}$$ and $$\text {RF}_\text {n, H}$$, we adopt $$\text {RF}_\text {n}$$ with $$\text {RF}_\text {n}$$ = $$\text {RF}_\text {n, L}$$ = 131.5 $$\cdot$$$$\text {RF}_\text {n, H}$$.

The relationship between RM and $\text {RF}_\text {n}$ in BFO memristive devices is depicted in Fig. 3. The modulation of $\text {RF}_\text {n}$ in the BFO model is achieved by adjusting the values of $k_\text {n}$ and $k_\text {p}$, which impact the leakage current values (Fig. 3a) and the hysteretic current values in the positive voltage ranges (Fig. 3b), respectively. By decreasing the value of $k_\text {n}$ or increasing the value of $k_\text {p}$, the $\text {RF}_\text {n}$ value can be incrementally increased.

As shown in Fig. 3c,d, $\text {RF}_\text {n}$ exhibits a consistent correlation with RM, unlike the on/off ratio and $\text {RF}_\text {p}$. Increasing the values of $\text {RF}_\text {n}$ through modifications of $k_\text {n}$ or $k_\text {p}$ consistently leads to an increase in RM. However, it is important to notice that the underlying mechanisms governing the observed trends in RM differ between $k_\text {n}$ and $k_\text {p}$. The variations in effective voltage $V_\text {eff}$ with respect to $\text {RF}_\text {n}$ (for both HRS$^w$ and LRS$^w$ writing schemes), as well as their corresponding equivalent circuits, are demonstrated, while varying $k_\text {n}$ (Fig. 3e,f) and $k_\text {p}$ (Fig. 3g,h), respectively. In these equivalent circuits, different resistance states of the selective cell and cells in three distinct regions are represented by different colors. For HRS$^w$ (Fig. 3e,g), the selected cell is in HRS (blue) and RG1/RG3 cells are in LRS (orange), while for LRS$^w$ (Fig. 3f,h), the selected cell is in LRS (orange) and RG1/RG3 cells are in HRS (blue). It should be noted that the RG2 cells, regardless of their initialization in different writing schemes, are represented in gray in all equivalent circuits since they are reverse biased. Additionally, the cells in RG3 are not biased due to their grounded TEs and BEs in the OneWLPU reading scheme. Owing to the voltage divider effect among $R_\text {PU}$ and memristive cells in passive crossbar array, the resistance values of RG1 ($R_\text {RG1}$), RG2 ($R_\text {RG2}$), and the selected cell ($R_\text {sel}$) are crucial in determining $V_\text {eff}$, which, in turn, impacts RM while maintaining a constant $R_\text {PU}$.

For instance, when $\text {RF}_\text {n}$ is increased by reducing $k_\text {n}$, there is a more significant increase in $V_\text {eff,HRS}$ compared to $V_\text {eff,LRS}$, resulting in a higher RM, as shown in Fig. 3e,f. This enhancement in $V_\text {eff,HRS}$ and $V_\text {eff,LRS}$ can be attributed to an increase in the total effective resistance ($R_\text {eff}$), which includes the resistance of the selected cell $R_\text {sel}$ and the parasitic resistances $R_\text {RG1}$ and $R_\text {RG2}$ in parallel, especially the increased resistance of $R_\text {RG2}$ is a result of decreased leakage current achieved by modifying $k_\text {n}$ from 1.0E−7 V to 1.0E−8 V. The reason behind this is that the increased reverse-biased resistance $R_\text {RG2}$ in RG2 induces a more pronounced variation in the $R_\text {eff}$ in HRS$^w$ when the selected cell in parallel connection is in HRS, compared to LRS$^w$ when the selected cell is in LRS. In contrast, when $k_\text {p}$ is altered, the rise in RM, as depicted in Fig. 3d, is due to a more substantial decrease in $V_\text {eff,LRS}$ (Fig. 3h) compared to $V_\text {eff,HRS}$ (Fig. 3g). This decrement in $V_\text {eff,HRS}$ and $V_\text {eff,LRS}$ can be attributed to a reduction in the resistances of $R_\text {sel}$ and $R_\text {RG1}$ achieved by modifying $k_\text {p}$ from 1.5E−8 V to 1.5E−7 V. The more pronounced decline in $V_\text {eff,LRS}$ compared to $V_\text {eff,HRS}$ can be explained by the fact that the reduction in the $R_\text {sel}$ in the LRS causes a more distinct reduction in $R_\text {eff}$ compared to the reduction in the $R_\text {sel}$ in the HRS.

The aforementioned results suggest that increasing $\text {RF}_\text {n}$ through adjustments in the leakage current in negative bias range by varying $k_\text {n}$ or in the hysteresis current in positive bias range by varying $k_\text {p}$ can effectively increase the RM. The $\text {RF}_\text {n}$, i.e. $\text {RF}_\text {n, L}$ and $\text {RF}_\text {n, H}$, proves to be a superior factor for characterizing the functional behavior of self-rectifying passive crossbar arrays, outperforming the commonly used on/off ratio and nonlinearity metrics found in state-of-the-art research.

Since the simulation studies in this work are based on the self-rectifying I-V characteristics of BFO memristors, the conclusion that negative rectification factors decisively influence the performance of self-rectifying passive crossbar arrays can be extended to other types of memristors and non-volatile memory (NVM) materials with self-rectifying I-V characteristics. NVM materials operate based on various principles: phase change memory (PCM) primarily utilizes GeSbTe and GeSe alloys, with a switching mechanism dependent on phase transitions from amorphous to crystalline states, altering electrical resistance for data storage^52,53^; spintronic materials, such as CoFeB, are utilized in magnetic random access memory (MRAM) through magnetic tunnel junction (MTJ) structures, where current pulses alter the magnetization of the free layer, resulting in two distinct resistance states for data storage^54,55^; ferroelectric materials, such as PbTiO_3_, SrTiO_3_, and Pb(ZrTi)O_3_, store data through the switchable polarization state of the ferroelectric material^56–58^; and resistive random access memory (RRAM) relies on the resistive switching effect of metal oxide materials, such as TaO_x_ and TiO_2_, due to the formation and rupture of conductive filaments^4,20,59^. PCM, spintronic materials, and ferroelectric materials lack the necessary self-rectifying properties and are thus unsuitable for passive crossbar arrays. In contrast, oxide-based RRAM materials, such as TiN/TiO_x_/HfO_x_/Au^60^ and Si/SiO_2_/Si^27^, exhibit a high negative rectification factor ($\text {RF}_\text {n, L}$) reaching 10^5^, making them ideal candidates for mitigating sneak-path problems in passive crossbar arrays.

**Fig. 4:**
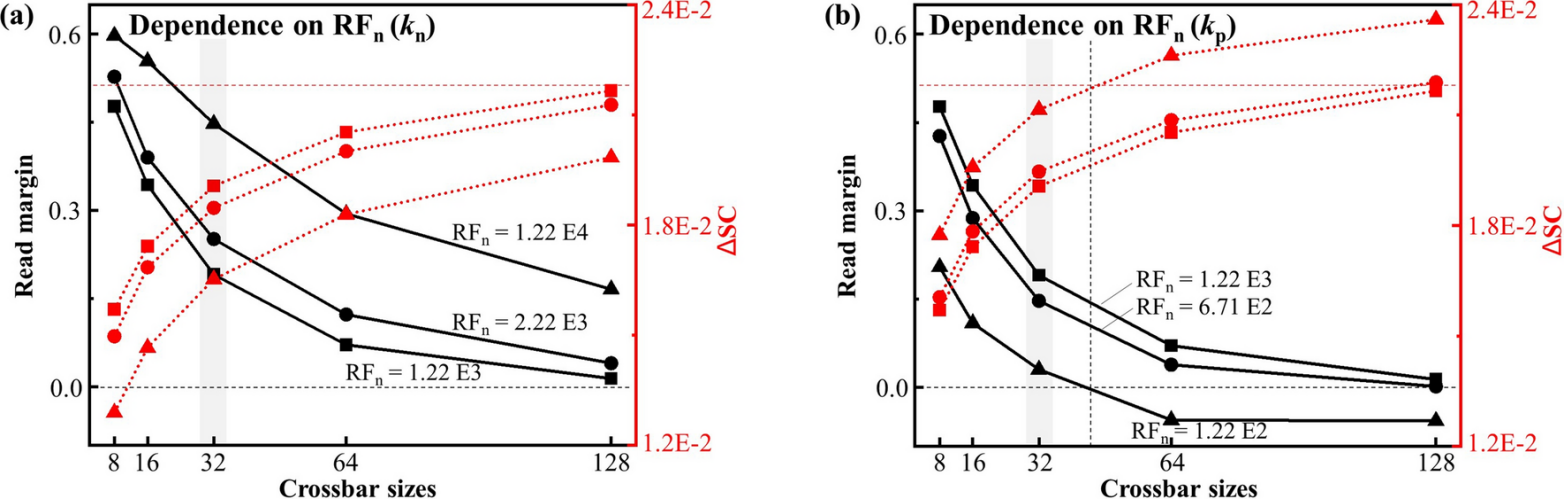
Evaluation of $\Delta$SC and RM with respect to crossbar sizes. The $\text {RF}_\text {n}$ is changed by modifying the (**a**) $k_\text {n}$ and (**b**) $k_\text {p}$ in the BFO model. The black dash line marks threshold value of RM = 0, while red dash line marks threshold value of $\Delta$SC = 2.19 E−2. The data points at crossbar size of 32 $\times$ 32, corresponding to Fig. 3c and Fig. 3d, are shadowed with gray back ground color.

### Sneak path current as performance identifier

The presence of the sneak path effect in crossbars, caused by the parasitic currents of RG1, RG2, and RG3, has a significant impact on $V_\text {eff}$ due to the voltage divider effect, thereby affecting RM. Establishing a direct correlation between RM and the parasitic currents in the crossbar array remains a formidable challenge, mainly due to the complexity of the sneak path effect. In this section, we conduct a comprehensive study on the sneak path effect using $\text {RF}_\text {n}$ as a basis, and based on the findings, we propose a quantitative link between the SC and RM.

In the Fig. 3e–h, the SCs through individual cell in regions RG1 and RG2 in the OneWLPU reading scheme are illustrated as red curves, while $\text {RF}_\text {n}$ is altered by parameters $k_\text {n}$ (Fig. 3e,f) and $k_\text {p}$ (Fig. 3g,h). It should be noted that the SC in OneWLPU reading scheme considers the parasitic current flows through series-connected forward-biased cells in RG1 and reverse-biased cells in RG2, thus the current trend is consistent among individual cells in RG1 and RG2. Additionally, both the TE and BE of the cells in RG3 are grounded, resulting in no observable current flowing through these cells. Although a consistent current trend is observed between RG1 and RG2 in each subfigure, there is no monotonic trend observed in the SCs when varying $k_\text {n}$ and $k_\text {p}$, which represent the change in leakage current in the negative bias range and the hysteresis current in the positive bias range, respectively.

For example, as depicted in Fig. 3e,f, the SCs via individual cells in both RG1 and RG2 in the HRS$^w$ and LRS$^w$ are reduced with an increment in $\text {RF}_\text {n}$ induced by a decrement in $k_\text {n}$, attributable to the decreased leakage current of reverse biased cells in RG2. In contrast, in Fig. 3g,h, the SCs through individual cells in RG1 and RG2 demonstrate an increase in HRS$^{w}$ and a decrease in LRS$^{w}$ as a result of the increasing $\text {RF}_\text {n}$ induced by the increment in $k_\text {p}$. This behavior can be attributed to the higher hysteresis current observed during the readout process when $k_\text {p}$ is increased in the positive bias range. In HRS$^{w}$, the increased SC is primarily caused by a more pronounced current increase in RG1 with LRS cells. On the other hand, in LRS$^{w}$, the decreased SC is due to the significant increase in current flow through the selected LRS cell, resulting in a reduction of SC current.

In order to evaluate the functional behavior of a passive crossbar array, the newly proposed metrics based on SCs should satisfy the following criteria: (1) They should align with the well-established relationship between SC and RM, which are qualitatively studied in previous work^4,28,61^, where an increase in RM generally corresponds to a decrease in SC in the passive crossbar, regardless of variations in $$k_\text {n}$$ and $$k_\text {p}$$. (2) They should capture the essential characteristics observed and discussed in RM, as depicted in Fig. 3c,d, while accounting for variations in $$k_\text {n}$$ and $$k_\text {p}$$. In accordance with these conditions, and based on the analysis of the observed relationship between RM and SCs in different regions in Fig. 3, we propose a new metric, i.e. $$\Delta$$SC, which is determined as follows for characterizing the functional behavior of a self-rectifying passive crossbar array ($$\text {RF}_\text {n, L}$$$$\gg$$ 1):

4 $$\begin{aligned} \Delta \text {SC} = \left\{ \begin{array}{ll} \frac{1}{\lg ({I_\text {LRS,RG2})} \cdot \lg ({I_\text {HRS,RG2})}}& \quad \text {RF}_\text {n, H}\ge 1\\ \frac{1}{\text {RF}_\text {n, H}} \cdot \frac{1}{\lg ({I_\text {LRS,RG2})} \cdot \lg ({I_\text {HRS,RG2})}},& \quad \text {RF}_\text {n, H}< 1 \end{array} \right. \end{aligned}$$

The $I_\text {HRS,RG2}$ and $I_\text {LRS,RG2}$ represent the total SCs flowing through RG2 in the OneWLPU reading scheme in the HRS$^w$ and LRS$^w$, respectively. $\Delta$SC can be described as dimensionless, as it is calculated using the logarithmic values of current in Eq. (4). The total SC is computed by multiplying the SC of an individual cell in RG2 (shown in Fig. 3e–h) with the number of cells in RG2, i.e., $(m-1)^2$, where *m* represents the dimensions of a *m*$\times$*m* crossbar. The calculation of total SC is applicable in this study since the variations in C2C and D2D of the memristive cells are not considered. Additionally, the line resistance of 10 $\Omega$, which is assumed in this work, does not result in recordable current differences among cells in the passive crossbar due to the high resistive operation of the BFO memristor in both HRS and LRS.

The computed $$\Delta$$SC, alongside the RM, is depicted in Fig. 3c and Fig. 3d, with varying $$k_\text {n}$$ and $$k_\text {p}$$, respectively. As observed, in general, a decreasing $$\Delta$$SC is observed with an increasing RM with respect to $$\text {RF}_\text {n}$$, regardless of varying $$k_\text {n}$$ or $$k_\text {p}$$. In both cases of $$k_\text {n}$$ and $$k_\text {p}$$, the RM demonstrates a linear increase accompanied by exponential growth of $$\text {RF}_\text {n}$$, and similarly, the $$\Delta$$SC exhibits a linear dependency on the exponential increase of $$\text {RF}_\text {n}$$, which validates the significant role of the $$\text {RF}_\text {n}$$ in affecting the SC and the RM. As another primary trend of the RM, the variations in $$k_\text {n}$$ exert a more pronounced influence on the changes in RM, compared to the variations in $$k_\text {p}$$, indicating more significant impact of the leakage current on the RM, in contrast to the hysteresis current in the positive bias range in $$I-V$$ characteristics of memristive devices. This feature is captured in $$\Delta$$SC too, and the reason is the multiplication of the total SCs in HRS$$^w$$ and LRS$$^w$$ in RG2, which especially reduces the slope of decrease in $$\Delta$$SC with respect to $$\text {RF}_\text {n}$$ while varying $$k_\text {p}$$ in comparison to the case of varying $$k_\text {n}$$. Furthermore, it is important to highlight that in this study, the SCs in RG2 are utilized to assess the $$\Delta$$SC, as the reverse biased cells in RG2 possess consistent and highest reverse bias resistance in both the HRS$$^w$$ and LRS$$^w$$ writing schemes, which restricts the overall SC in these regions, especially if $$\text {RF}_\text {n, H}$$$$\ge$$ 1. If $$\text {RF}_\text {n, H} < 1$$, where $$R_\text {RG1}>$$$$R_\text {RG2}$$, the SC under LRS$$^w$$ writing scheme is restricted by $$R_\text {RG1}$$ instead of $$R_\text {RG2}$$. Hence, the influence by $$\text {RF}_\text {n, H}$$ shall be considered for computing $$\Delta$$SC by multiplying a factor 1/$$\text {RF}_\text {n, H}$$ as shown in Eq. (4). For example, as depicted in Fig. 4b, in the case of $$\text {RF}_\text {n}$$ = 1.22E2, where the $$\text {RF}_\text {n, H}$$ = 9.27E−1 < 1, the $$\Delta$$SC is computed with considering $$\text {RF}_\text {n, H}$$.

To further validate the effectiveness of $\Delta$SC as a metric for assessing valid functional behavior of passive crossbar array, we examine the $\Delta$SC with respect to crossbar size of 128 $\times$ 128 (16K), in comparison to RM (Fig. 4). The corresponding data with crossbar size of 32 $\times$ 32 which were demonstrated in Fig. 3 are marked with a gray back ground color in Fig. 4. The results in Fig. 4 demonstrate that as the crossbar size increases, an increase in $\Delta$SC can be observed, accompanying a decrease in RM. With each crossbar size, a higher $\text {RF}_\text {n}$ value empowers the passive crossbar array with an improved performance assessed by a lower $\Delta$SC or a higher RM. Especially, Fig. 4a shows, at individual crossbar size, more significant RM increasement can be gained at $\text {RF}_\text {n}$ value of 1.22E4, in comparison to the $\text {RF}_\text {n}$ values of 1.22E3 or 2.22E3. This phenomenon is captured by a more prominant decrease in $\Delta$SC, as $\Delta$SC maintains a mirrored reversed relationship with RM. Moreover, in Fig. 4b, for a given $\text {RF}_\text {n}$ value (e.g., $\text {RF}_\text {n}$ = 1.22E2), the intersection point of the red dashed line (representing the threshold value of $\Delta$SC = 2.19E−2) with $\Delta$SC and the intersection point of the black dashed line (representing the threshold value of RM = 0) with RM perfectly coincide at the same crossbar size. This implies that $\Delta$SC = 2.19E−2, which is comparable to RM = 0, can serve as a valid quantitative metric for evaluating the functional characteristics of a passive crossbar array. When $\Delta$SC < 2.19E−2 (RM > 0), it indicates the presence of a distinct reading window between LRS and HRS in the selected cell during operation, confirming the valid functional behavior of the BFO-based self-rectifying passive crossbar array.

The results presented in this study confirm that $\Delta$SC represents a novel and reliable quantitative metric for capturing the characteristics of RM across different crossbar sizes, including up to 16K. Unlike RM, $\Delta$SC does not require the inclusion of $R_\text {PU}$ in the analysis of the voltage divider effect between $R_\text {PU}$ and the memristive passive crossbar, thereby eliminating the dependence of crossbar performance evaluation on the selection of $R_\text {PU}$.

## Conclusion

In conclusion, our comprehensive analysis has unraveled the intricate relationship between the RM and the sneak path effect within self-rectifying passive crossbar arrays. By scrutinizing relevant parameters traditionally used to characterize self-rectifying behavior in memristive cells, we have revealed the limitations of conventional metrics like the on/off ratio and nonlinearity in providing a holistic understanding of functional performance within passive crossbar arrays. These metrics, we assert, are ill-suited for quantitatively establishing the intricate interplay between RM and parasitic sneak path current, primarily because they fail to capture the rectifying features essential within the reversed bias region. Consequently, we have proposed and validated the use of a pair of negative rectification factors, $\text {RF}_\text {n, L}$ and $\text {RF}_\text {n, H}$, applicable to both the LRS and HRS, as performance metrics that surpass traditional measures like the on/off ratio and nonlinearity. Building upon these findings, we introduced a novel performance identifier, $\Delta$SC, which harnesses the sneak path effect to accurately evaluate the functional behavior of passive crossbar arrays without the need for an external $R_\text {PU}$. Through simulation implementation using BFO memristive cells with negative rectification factors, specifically $\text {RF}_\text {n, L}$ = 1.22E3 and $\text {RF}_\text {n, H}$ = 9.27, we have successfully established a quantitative link between RM and sneak path current. This effort has resulted in the validation of the functional performance of the passive crossbar array, achieving $\Delta$SC values < 2.19E−2, corresponding to RM > 0, across various array sizes up to 16K.

Looking ahead, this work underscores the potential of emerging passive crossbar arrays based on self-rectifying memristive cells, which offer cost advantages in terms of area and power when compared to traditional 1T1R configurations. Furthermore, it is important to mention the inherent non-idealities of memristive devices, such as device variations, which can limit the functional behavior of passive crossbars and impact the practical implementation of memristive technology, and shall be further studied as a possible future work. Additionally, while our analysis focused on the floating writing scheme and the OneWLPU reading scheme, which are widely used for practical testing of memristive crossbars due to their ability to suppress sneak path current^26,43^, various writing/reading schemes, such as 1/2 or 1/3 writing/reading schemes, can further suppress sneak path current and significantly influence the functional behavior of passive crossbar arrays^26,62–64^. In our future work, we plan to evaluate the functional performance of passive crossbar arrays under these schemes while incorporating the positive rectification factor into the $\Delta$SC equation, continuing our efforts to advance the efficient characterization of functional performance of memristive passive crossbars and their impact on future practical applications.

## Methods

### Experiments

The polycrystalline BFO thin film with the thickness of 500 nm was deposited by pulsed laser deposition (PLD) on a Pt/Ti$$/$$$$\textrm{SiO}_2$$$$/$$Si substrate with 100 nm/50 nm thick Pt/Ti layer^65^. Circular Au top electrodes with an area of $$10^5 ~\upmu \textrm{m}^2$$ and a thickness of 150 nm were fabricated by DC magnetron sputtering at room temperature using a metal shadow mask. All the electrical measurements presented in this study were conducted using a Keithley source meter 2400, which was connected to a PC via GPIB cables and controlled through LabVIEW program.

### Simulations

The mathematical model of BFO memristor has been established and is represented by three equations in Supplementary Fig. S1. These mathematical equations were subsequently transformed into Verilog-A code for simulation purposes in Cadence Virtuoso, to study the application of BFO memristor-based crossbar arrays. To operate the crossbar array, three operation cycles were applied, and each cycle lasted for 100 ms during the simulations.

## Supplementary Information

Supplementary Information.

## Data Availability

The datasets used and/or analyzed during the current study are available from the corresponding author on reasonable request.
