# Supplementary material for: Assessment of functional performance in self-rectifying passive crossbar arrays utilizing sneak path current
Source: Sci Rep. 2024 Oct 21;14:24682. doi: 10.1038/s41598-024-74667-z (PMC11494113; doi:10.1038/s41598-024-74667-z)
Supplement: Supplementary file 1 — Supplementary Information. [file 41598_2024_74667_MOESM1_ESM.docx]

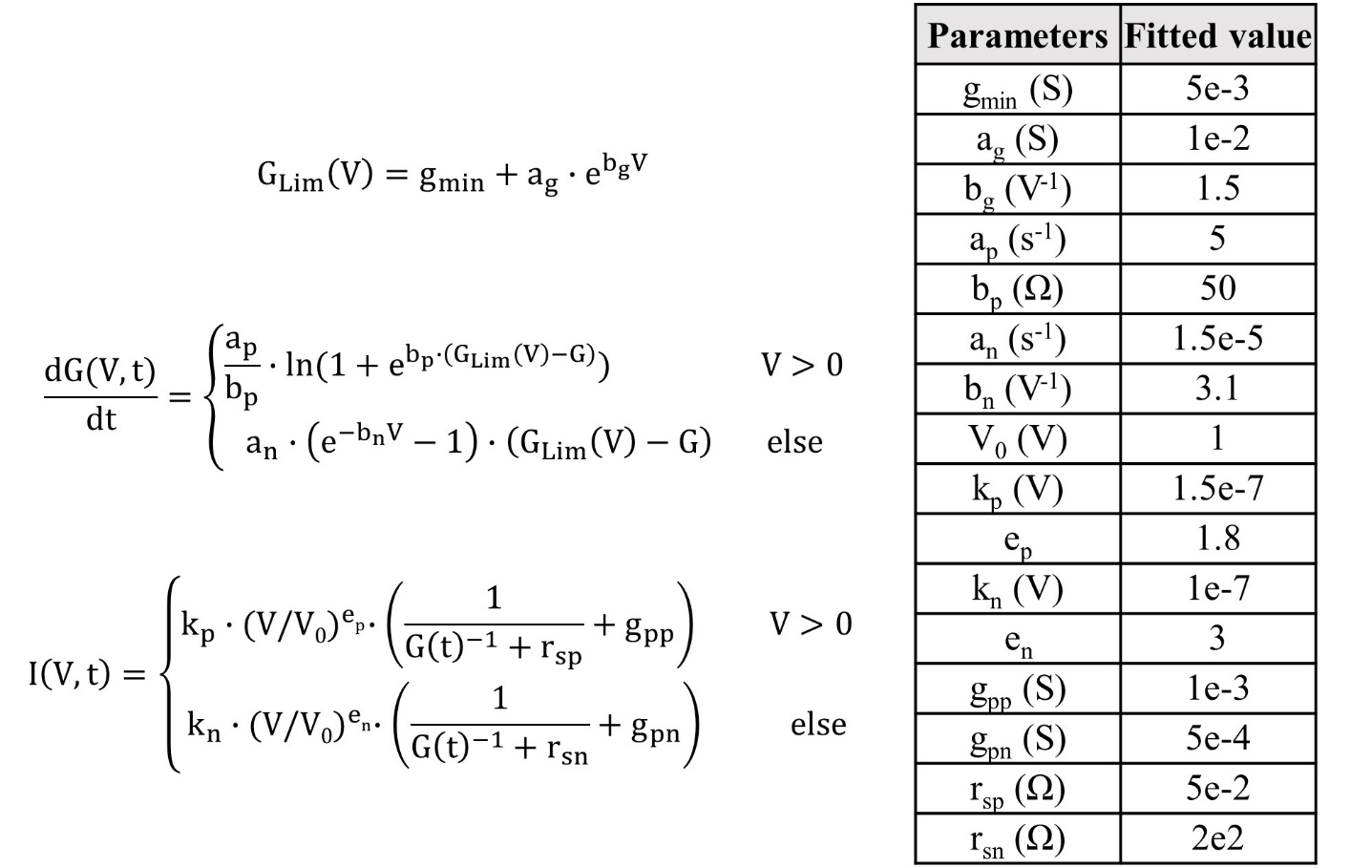


Figure S1: Behavioral mathematical model for the BFO memristive device. The simulation parameters are given in the corresponding tables as insets.
